#  Study of the Effects of ATP Suppliers and Thiol Reductants on Toxicity of Pioglitazone in Isolated Rat Liver Mitochondria 

**Published:** 2015

**Authors:** Abbas Rezaiean Mehrabadi, Akram Jamshidzadeh, Marzieh Rashedinia, Hossein Niknahad

**Affiliations:** Department of Pharmacology and Toxicology, Faculty of Pharmacy, Shiraz University of Medical Sciences, Shiraz, Iran.

**Keywords:** ATP suppliers, Mitochondria, Pioglitazone, Toxicity

## Abstract

Pioglitazone (PG) is one of thiazolidinediones used for the treatment of type II diabetes mellitus. Some reports of its hepatotoxicity exist, but the mechanism of its hepatotoxicity is not well known. In the present study, the protective effect of some ATP suppliers are investigated against mitochondrial toxicity of PG in isolated rat mitochondria. Mitochondrial viability was investigated by MTT assay. The effects of PG on superoxide dismutase activity, ATP production, mitochondrial swelling and oxidative stress were also investigated. PG reduced mitochondrial viability with an LC_50_ of 880±32 µM. It reduced ATP production and superoxide dismutase activity in mitochondria and increased mitochondrial swelling, but no oxidant effect was present as measured by TBARS formation. Fructose, dihydroxyacetone, dithioteritol, and N-acetylcysteine reduced mitochondrial toxicity of PG. Therefore, PG toxicity may be due to its mitochondrial toxicity and energy depletion, and ATP suppliers could be effective in preventing its toxicity.

## Introduction

Thiazolidinediones (TZDs), fibrates, and statins show a variety of negative pharmaco-toxicological profiles and some TZDs cause hepatotoxicity (1). Troglitazone, a thiazolidinedione, was introduced as a promising antidiabetic drug, but it had to be withdrawn from the market within a few years because of its severe hepatotoxicity induced by inhibition of bile salt transport from hepatocytes, apparently by its sulfate conjugate (2). 

Development of darglitazone and ciglitazone was discontinued, with the latter showing cataractogenic potential in rats (3). The development of muraglitazar, the first PPAR dual agonist, was dropped because of increased cardiotoxicity (4). Reported hepatotoxicity cases involving the newer TZDs, rosiglitazone and pioglitazone, have been few in number and less severe in consequence. Six cases of rosiglitazone-induced hepatotoxicity and 5 of pioglitazone-induced hepatotoxicity have been reported. Most patients improved symptomatically 2 to 4 weeks following discontinuation of the offending TZDs, with normalization of liver enzyme levels in 2 weeks to 6 months following TZDs discontinuation (5). Pioglitazone has a reactive ring-opened product which has been identified in rat and human liver microsomes and in freshly isolated rat but not human hepatocytes (6). It is trapped by glutathione and can be a cause of hepatotoxicity.

Mitochondrial impairment is involved in the etiology of hepatoxicity, myopathy, cardiomyopathy, rhabdomyolysis, and other side effects of some contemporary therapeutics (7). 

Some studies suggest that TZDs-induce mitochondrial swelling, disturbance in membrane potential and abnormality in mitochondrial permeability. TZDs also behaved as uncouplers of respiration in addition to having strong inhibitory effects on Complexes II^+^, III, IV, and V in mitochondrial respiratory chain (8), and troglitazone is cytotoxic to HepG2 cells and rat and human hepatocytes (9). 

Mitochondrial swelling can be initiated by many agents and spontaneous swelling can be reversed by the addition of adenosine triphosphate (10). Mitochondrial depolarization and swelling can also be due to non-specific permeabilization of the inner mitochondrial membrane (11).

The purpose of this study was to investigate the mechanisms of pioglitazone toxicity and protective effects of some ATP suppliers such as fructose and dihydroxyacetone and thiol reductant e.g dithioteritol and N-acetylsysteine.

## Experimental

*Chemicals*

Pioglitazone hydrochloride, 5-diphenyl tetrazolium bromide (MTT), dithioteritol (DTT), N-acetylcysteine (NAC), and other chemicals were purchased from the Sigma Chemical Co. (Germany). Dihydroxyacetone (DHA), fructose and 5,5'-dithiobis-(2-nitrobenzoic acid (DTNB) were purchased from Merck Chemical Co. (Germany).

*Animals*

Male Sprague-Dawley rats (200-250 g) were obtained form the Laboratory Animals Research Center of Shiraz University of Medical Sciences. The rats were maintained under controlled temperature, 12 hours light/12 hours dark conditions for one week before the start of the experiments. They were allowed to feed standard laboratory chaw and tap water *ad libitum*. The animals were treated according to the guideline of the Ethics Committee of Shiraz University of Medical Sciences.

*Preparation of mitochondria*

Rats were anaesthetized by injection of 60 mg/Kg thiopental and the liver was removed and minced in a cold manitol solution containing 0.225 M D-manitol, 75 mM sucrose and 0.2 mM ethylenediaminetetraacetic acid (EDTA). Approximately 30 g of the minced liver was gently homogenized in a glass homogenizer with a Teflon pestle and then centrifuged at 700×*g* for 10 min at 4 °C to remove nuclei, unbroken cells and other non-subcellular tissues. The supernatant was centrifuged at 7000×*g* for 20 min. The second supernatant was pooled as the crude microsomal fraction and the pale loose upper layer, which was rich in swollen or broken mitochondria and lysosomes was washed away. The dark packed lower layer (heavy mitochondrial fraction) was resuspended in the manitol solution and recentrifuged twice at 7000×*g* for 20 min. The heavy mitochondrial sediments were suspended in Tris solution containing 0.05 M Tris-HCl buffer (pH 7.4) 0.25 M sucrose, 20 mM KCl, 2.0 mM MgCl_2_ and 1.0 mM Na_2_HPO_4_ at 4 °C before assay.

*Experimental design*

In order to determine the LC_50_ of pioglitazone, mitochondrial viability was investigated by 3-(4, 5-dimethylthiazol-2-yl) 2, 5-diphenyl tetrazolium bromide (MTT) assay. Different concentrations of pioglitazone (10 to 1000 µM) were used and LC_50_ of pioglitazone was determined.

In the rest of the study, a dose of 880 µM pioglitazone with regards to LC_50_, and a submaximal dose of DTT, NAC, fructose or DHA with regards to pervious studies were selected. Mitochondrial suspensions were divided into 10 groups of six duplicate samples. Group 1 was considered as control, groups 2 to 5 were treated with DTT, NAC, fructose and dihydroxyacetone, group 6 treated with pioglitazone (880 µM) and group 7 to 10 treated with pioglitazone (880 µM) plus DTT, NAC, fructose and dihydroxyacetone, respectively. Mitochondrial suspensions were incubated for 1 hour at 37 °C.

*MTT assay*

Viability of isolated mitochondria was assessed by a modified MTT assay (12). Tubes containing mitochondria were incubated at 37 ˚C for 1 h. After washing with the mitochondria suspension buffer, they were centrifuged at 1000 g and 4 ˚C for 20 min. Then 1 mL of MTT solution was added to each tube and incubated at 37 ˚C for 1 h. After centrifugation at 1000 g for 20 min again, 1 mL DMSO was added to each tube and the tubes were shaked well. Then, 100 µl of each sample was poured in Elisa plate wells, and the intensity of color was measured by Elisa reader at 560 and 630 nm. Viability of mitochondria was measured as a percentage of control (12).

*Lipid peroxidation*

Lipid peroxidation was assessed by measurement of thiobarbituric acid reactive compounds (TBARS). The amount of reactive products formed was calculated by using an extension coefficient of 165 mM^-1^ cm^-1^ at 532 nm (13).

*Determination of GSH and GSSG*

The supernatant was analyzed for reduced glutathione (GSH) by the 5, 5'-dithiobis-2-nitrobenzoic acid (DTNB) recycling procedure (14). GSSG (oxidized glutathione) plus GSH were determined in supernatant after mixing with 1 mL of 5% sodium borohydride (NaBH_4_), a reducing agent, and the resulting sulfhydryl groups of GSH were assayed as described (15).

*Superoxide dismutase activity*

Superoxide dismutase (SOD) activity was determined using a Cayman Chemical SOD assay Kit. SOD catalyzes the dismutation of superoxide anion radical (O_2_^˚−^) to hydrogen peroxide and oxygen. In the presence of SOD, the superoxide radical O_2_^˚−^ undergoes a dismutation into O_2_ and H_2_O_2_, which results in formazan formation. Hence, this competing assay yields to the indirect measurement of SOD activity (16,17). One hour after incubation of mitochondria with pioglitazone alone or with other compounds, the suspension was washed twice with buffer B, the 1 mL de-ionized water was added and centrifuged. Ten mL of the sample was added to microplates and 200 µL of radical detector was added. In the last stage, 20 µL of xanthine oxidase was added and after shaking and equilibrating with the room temperature, its absorbance was monitored at 400-460 nm. 

 SOD activity of the samples was calculated using the equation from the linear regression of the standard curve for each sample. One unit is defined as the amount of enzyme needed to exhibit 50% dismutation of the superoxide radical (16,17).

*Protein measurement*

Mitochondrial protein concentrations were determined using the method developed by Bradford (18).

*Mitochondrial swelling measurement*

 Two mL of sucrose – Tris solution (0.34 M) was added to 1 mL of mitochondria suspension. The absorbance was recorded every 5 min for 1 hour, at 250 nm. One mL of sucrose–Tris solution (0.34M) was as blank. Then one mL of the solution containing: MgCl_2_ 0.003 M, ATP 0.005 M, EDTA 0.2 mM was added and again the absorbance was recorded at 250 nm.

*Measurement of ATP*

 ATP levels were measured by HPLC, using a C-18 column, Knouver HPLC system coupled with a dual absorbance UV detector (Model 2487) equipped with a computer system with a ChromGate software program for data processing, as previously explained (19). Briefly, one mL of isolated mitochondria was mixed with 1 mL of KOH 0.05 M (on ice), then, 2 mL deionized water was added; after 2 min, 650 μL of KH_2_PO_4_ (0.05 M) was added, and vortexed. After filteration using filter paper, this solution was kept in freezer until injection to HPLC. ATP was measured by the method explained before. The mobile phase was 0.05 M ammonium dihydrogen phosphate (pH=6.0) with a flow rate of 1 mL/min. The absorbance of nucleotides was recorded at 254 nm and the run time of 20 min. The concentration of ATP was determined in terms of nanomole nucleotide per mg of protein.

*Statistical analysis*

All values were expressed as Mean±SEM of 6 experiments. Analysis of variance (ANOVA) followed by student Newmans-Keuls test was used to evaluate the significance of the results obtained. All analyses were performed using SPSS software.

## Results

*Effect of pioglitazone on the viability of mitochondria*

**Figure 1:**
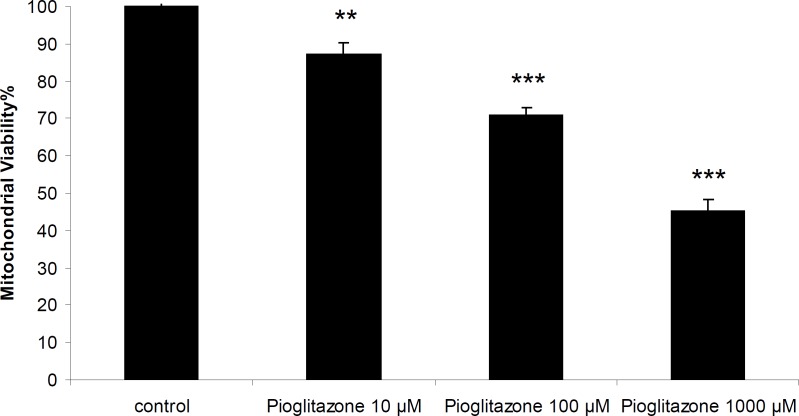
Mitochondrial viability in the presence of different concentrations of pioglitazone.

Pioglitazone was toxic towards freshly isolated rat mitochondria and the incubation of mitochondria with pioglitazone for 1 hour resulted in loss of mitochondrial viability in a dose-dependent manner with an LC_50_ of 880 µM (846.59-910.97) (Figure 1). 

**Significantly different from control (p<0.01).

***Significantly different from pioglitazone (p<0.001).

**Figure 2:**
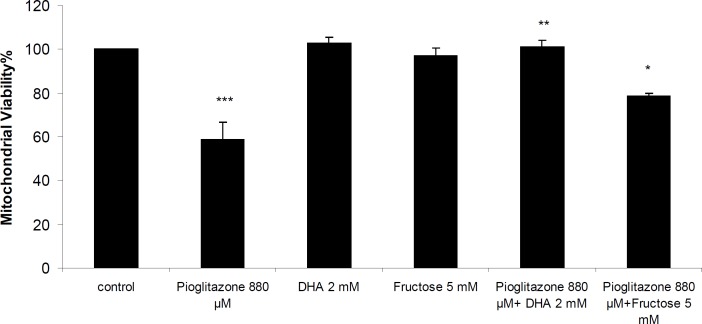
Mitochondrial viability in the presence of pioglitazone and DHA (2 mM) and fructose (5 mM). ***Significantly different from control (p< 0.001).**Significantly different from pioglitazone treated (*p*< 0.01). *Significantly different from pioglitazone treated (*p*<0.05).

**Figure 3:**
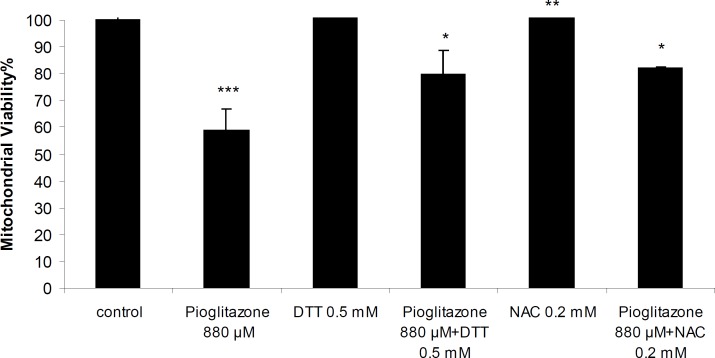
Mitochondrial viability in the presence of pioglitazone and DTT (0.5 mM) and NAC (0.2 mM). ***Significantly different from control (*p*< 0.001). **Significantly different from pioglitazone treated (*p*<0.01). *Significantly different from pioglitazone treated (p<0.05).

Simultaneous treatment of mitochondria with pioglitazone and dihydroxyacetone (DHA; 2 mM) or fructose (5 mM) significantly prevented pioglitazone toxicity (Figure 2). N-acetylcysteine (NAC; 0.2 mM), or dithioteritol (DTT; 0.5 mM) also significantly prevented the mitochondrial toxicity induced by pioglitazone (Figure 3).

*Effect of pioglitazone on GSH and GSSG content of mitochondria*

After one hour of incubation, GSH and GSSG content of pioglitazone-treated mitochondria did not change compared to untreated mitochondria (Table 1).

**Table 1:** Effect of pioglitazone on GSH/GSSG content of isolated rat liver mitochondria.

	**GSH (nmol/mg protein)**	**GSSG (nmol/mg protein)**
Control	26.60±1.96	3.45±0.23
Pioglitazone 880 μM	27.58±1.22	4.0±0.29

Note: GSH and GSSG were measured as explained under materials and methods.

All data are given as Mean±SEM of different experiments. pioglitazone did not affect on GSH/GSSG content of mitochondria compared to control.

Pioglitazone did not affect lipid peroxidation in isolated mitochondria and TBARs concentration was not significantly greater in mitochondrial suspension treated with pioglitazone than that of control non-treated mitochondria (Table 2).

**Table 2:** Effect of pioglitazone on lipid peroxidation in isolated rat liver mitochondria.

	**GSH (nmol/mg protein)**
Control	2.24±0.07
H_2_O_2_ 90 mM	3.20* ± 0.04
Pioglitazone 880 μM	1.92±0.02

Note: All data are given as Mean±SEM of different experiments.

TBARs concentration was not significantly greater in mitochondrial

suspension treated with pioglitazone than that of controls.

* significantly different from control (*p*<0.001).

*Effect of pioglitazone on mitochondrial swelling*

**Figure 4:**
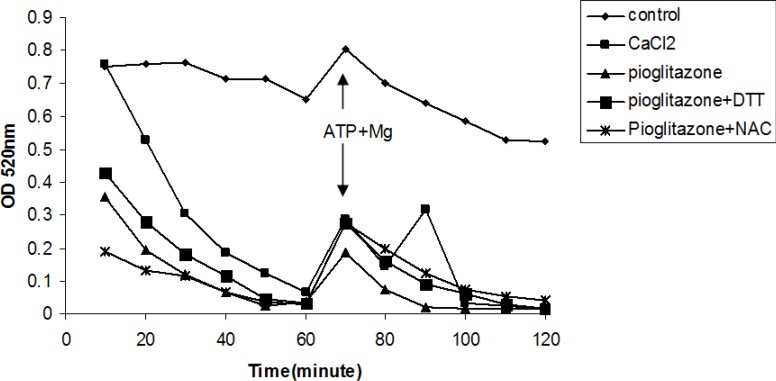
Effects of thiol reductants on mitochondrial swelling induced by pioglitazone and CaCl_2_.

 Pioglitazone (880 µM) induced mitochondrial swelling (Figure 4), however, DTT and NAC as thiol reductants (Figure 4), and fructose and DHA as ATP supplier did not prevent mitochondrial swelling induced by pioglitazone (data not shown).

*Effect of pioglitazone and ATP suppliers and thiol-reductants on SOD activity*

 Pioglitazone (880 μM) reduced SOD activity and DTT and DHA, but not fructose or NAC, prevented pioglitazone induced inhibition of SOD activity (Table 3).

**Table 3:** Effect of pioglitazone with and without ATP suppliers or thiol reductants on SOD activity in isolated rat liver mitochondria

	**SOD(U/mL/mg protein)**
Control	
Pioglitazone	79.11±3.51
DTT	58.19±2.69
Pio+DTT	74.33±0.86
DHA	73.25*±0.84
Pio+DHA	73.18±1.23
NAC	75.09*±1.70
Pio+NAC	79.26±0.93
Fructose	78.27±0.92
Pio+Fructose	76.33±1.43

Note: All data are given as Mean±SEM of different experiments.

Pio: Pioglitazone

DTT: Dithioteritol

DHA: Dihydroxyacetone

* Significantly different from pioglitazone *(p<*0.05).

*Effect of pioglitazone on ATP synthesis by mitochondria*

**Figure 5:**
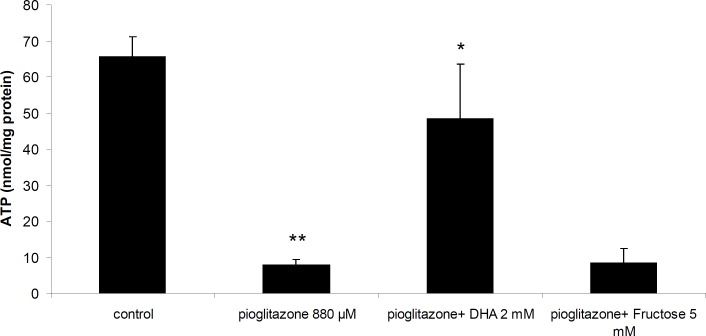
Effect of pioglitazone and DHA (2 mM) on ATP synthesis by mitochondria. **Significantly different from control (*p*< 0.01). *Significantly different from pioglitazone treated (*p*<0.05

 Incubation of mitochondria with 880 μM pioglitazone for 1 hour resulted in reduction of ATP synthesis by mitochondria. Addition of DHA (2 mM) to the incubation mixture prevented ATP depletion by pioglitazone (Figure 5). However, fructose (Figure 5), DTT or NAC were not able to prevent ATP depletion by pioglitazone (data not shown).

## Discussion

Thiazolidinediones (TZDs) act to decrease insulin resistance. Their primary action is the regulation of genes involved in glucose and lipid metabolism and adipocyte differentiation. TZDs are ligands of peroxisome proliferator-activated receptor-gamma (PPAR-γ), part of the steroid and thyroid superfamily of nuclear receptors. These PPAR receptors are found in muscle, fat, and liver. Pioglitazone and rosiglitazone are TZDs available currently in many countries. Pioglitazone has PPAR-α as well as PPAR-γ activity. Pioglitazone is metabolized by CYP2C8 and CYP3A4 to active metabolites (20). 

 Mitochondrial dysfunction caused by some of the TZDs, fibrates, and statins have been previously reported based on studies of membrane potential, mitochondrial swelling and assays of the respiratory chain in isolated mitochondria (21).

 Tirmenstein *et al.* showed that troglitazone caused a drop in membrane potential in HepG2 cells and also a decrease in ATP levels (22).

Masubuchi *et al*., have reported that troglitazone and ciglitazone, at concentrations of 25 μM and above, caused mitochondrial swelling, decreased mitochondrial membrane potential, and induced irreversible mitochondrial permeability transition in mouse liver mitochondria whereas rosiglitazone and pioglitazone did not cause these effects (21).

 In the present study, we showed that pioglitazone was toxic towards isolated rat liver mitochondria in a dose dependent manner, and decreased ATP synthesis by mitochondria. ATP supplier compounds reduced mitochondrial toxicity and prevented ATP depletion by pioglitazone. Therefore, the results of our study suggest that toxicity of pioglitazone is partly mediated by ATP depletion due to inhibition of mitochondrial respiration which supports previous reports (8). Our data also suggest that ATP suppliers are effective in preventing pioglitazone toxicity by providing energy. DHA was also able to bind pioglitazone (data not shown) *in-vitro* and possibly can prevent mitochondrial toxicity by removing pioglitazone from binding sites. 

Another mechanism proposed for toxicity of pioglitazone is oxidative stress and GSH depletion (7). However, pioglitazone did not induce lipid peroxidation or GSH depletion in mitochondria in our experiments which may be because of low metabolism or lack of metabolism of pioglitazone in isolated mitochondria. However, thiol providers, DTT or NAC protected mitochondrial toxicity of pioglitazone. Some studies have shown that pioglitazone has a reactive ring-opened product which is trapped by glutathione and positively identified by high performance liquid chromatography (7). Therefore, DTT or NAC possibly bind to pioglitazone metabolite or increase GSH content of mitochondria and detoxify the toxic metabolite(s) of pioglitazone. Therefore, it is possible that in our study because of lack of activity of metabolizing enzymes, as we used isolated mitochondria, the ring-opened product is not formed and GSH is not consumed.

Superoxide dismutases (SODs) are metalloenzymes that catalyze the dismutation of the superoxide anion to molecular oxygen and hydrogen peroxide and thus form a crucial part of the cellular antioxidant defense mechanisms (23).

Mitochondrial Mn-SOD prevents cellular damage because it scavenges toxic superoxide radicals (24). We found that the activity of SOD in liver mitochondria was inhibited significantly after 1 hour of pioglitazone treatment. This effect was prevented significantly by administration of DHA and DTT. However, because lipid peroxidation and GSH depletion were not present in this model, oxidative stress was not a major mechanism of toxicity of pioglitazone in isolated mitochondria.

Our results therefore, suggest that mitochondrial toxicity of pioglitazone is partially mediated by ATP depletion, and ATP suppliers such as DHA may provide a protective effect on pioglitazone-induced toxicity.
